# Modulation of PTH1R signaling by an ECD binding antibody results in inhibition of β-arrestin 2 coupling

**DOI:** 10.1038/s41598-019-51016-z

**Published:** 2019-10-08

**Authors:** Kaushik Sarkar, Lisa Joedicke, Marta Westwood, Rebecca Burnley, Michael Wright, David McMillan, Bernadette Byrne

**Affiliations:** 1 Department of Life Sciences, Imperial College, London, SW7 2AZ United Kingdom; 2 UCB Pharma, 208 Bath Road, Slough, SL1 3WE United Kingdom; 3 Present Address: M3, Western Avenue, Milton Park, Abingdon, Oxfordshire OX14 4SH United Kingdom; 4 Present Address: Vertex Pharmaceuticals, 86-88 Jubilee Avenue, Milton Park, Abingdon, Oxfordshire OX14 4RW United Kingdom

**Keywords:** Biochemistry, Biotechnology

## Abstract

Parathyroid hormone receptor 1 (PTH1R) belongs to the secretin class of G protein coupled receptors (GPCRs) and natively binds parathyroid hormone (PTH) and parathyroid hormone related peptide (PTHrP). Ligand binding to PTH1R involves binding to the large extracellular domain (ECD) and the orthosteric pocket, inducing conformational changes in the transmembrane domain and receptor activation. PTH1R regulates bone metabolism, signaling mainly through G_s_ and G_q/11_ G-proteins. Here, we used phage display to generate PTH1R ECD-specific antibodies with the aim of modulating receptor functionality. We identified ECD-scFvhFc, which exhibited high affinity binding to both the isolated ECD and to the full-length receptor in styrene-maleic acid (SMA) lipid particles. Epitope mapping using hydrogen-deuterium exchange mass spectrometry (HDX-MS) indicates that the α1 helix of the ECD is ECD-scFvhFc’s epitope which may partially overlap with the known PTH (1–34) binding site. However, PTH (1–34)-mediated G_s_ activation is Undisturbed by ECD-scFvhFc binding. In contrast, ECD-scFvhFc potently inhibits β-arrestin-2 recruitment after PTH (1–34)-driven receptor activation and thus represents the first monoclonal antibody to selectively inhibit distinct PTH1R signaling pathways. Given the complexity of PTH1R signaling and the emerging importance of biased GPCR activation in drug development, ECD-scFvhFc could be a valuable tool to study PTH1R signaling bias.

## Introduction

G-protein coupled receptors (GPCRs) represent one of the largest and most diverse membrane protein families, containing more than 800 members^1^. The importance of GPCR signaling is highlighted by the fact that approximately 34% of all currently prescribed drugs target GPCRs^2^. The receptors are classified according to sequence conservation and can be grouped into five distinct classes, including the secretin family of receptors. Secretin class receptors are characterized by the presence of a large extracellular domain (ECD) and are activated by peptide ligands engaging both the ECD and the transmembrane domain of the receptor^1,3^. The parathyroid hormone receptor 1 (PTH1R) is a well-characterized secretin class receptor involved in bone development and bone cell differentiation, and normally activated by parathyroid hormone (PTH) and parathyroid hormone-related peptide (PTHrP)^4–7^. Canonical GPCR signaling involves ligand binding which causes a conformational change in the transmembrane bundle and activation of the receptor^8^. This allows the coupling of a heterotrimeric G protein^9^ and the subsequent activation of a distinct cellular signaling pathway^10^. GPCR signaling is controlled by the coupling of β-arrestins which causes internalization of the receptor and inhibits further G protein signaling^11^. In recent years, research has revealed that the internalized β-arrestin-GPCR complex can signal through G protein-independent pathways including mitogen-activated protein kinases (MAPK), extracellular signal–regulated kinases (ERK), c-Jun N-terminal kinase (JNK), and p38 as well as Akt, PI3 kinase, and RhoA^12^.

**Figure 1:**
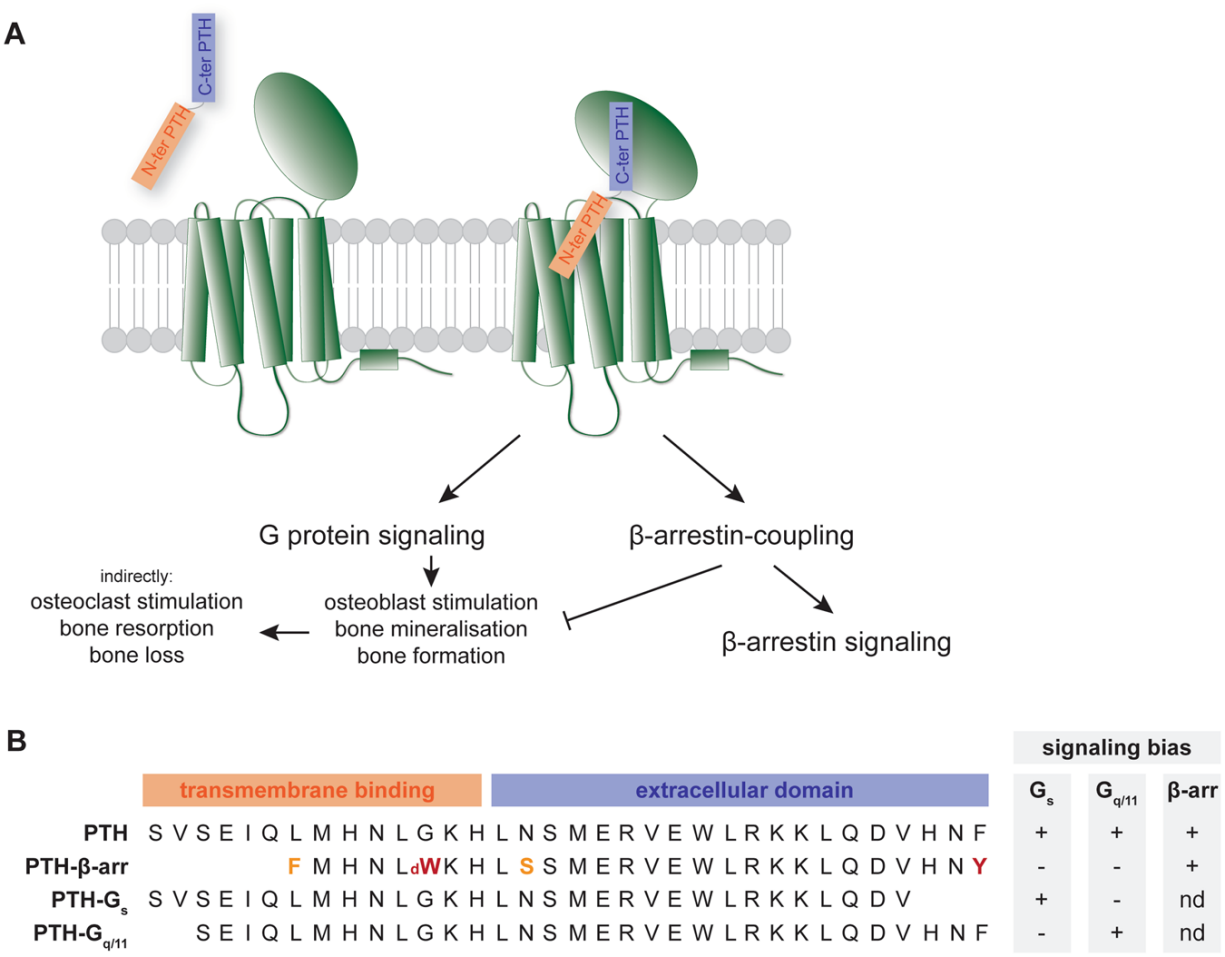
Signaling of PTH via the PTH1R is complex and triggers various signaling outcomes. (**A**) PTH binding to the PTH1R is bimodal and requires binding of the C-terminal fragment of the peptide to the extracellular domain of the receptor while the N-terminal part engages the transmembrane domain of the receptor. (**B**) Slight modifications of either the N- or C-termini of the peptides or introduction of a limited number of amino acid substitutions change the signaling outcome of the PTH1R. PTH-βArr is a modified bovine PTH, substitutions to the equivalent amino acid residue in human PTH are highlighted in orange, substitutions to a non-standard amino acid are shown in red. Abbreviations: dW, d-Tryptophan: nd, no data.

In the case of PTH1R, signaling has been described both by activation of G-protein dependent and independent pathways and a multitude of peptide ligand variants has allowed an in-depth characterization of the signaling behavior of the receptor (Fig. 1). PTH binding to PTH1R triggers coupling of the receptor to G_s_ and G_q/11_ generally resulting in osteoblast stimulation, bone mineralization and eventually bone formation^13^. However, prolonged PTH signaling causes bone resorption and bone loss through recruitment and activation of osteoclasts^14,15^. PTH-mediated G-protein signaling is normally terminated by recruitment of β-arrestin-mediated internalization maintaining a balance between bone formation and resorption^16^ (Fig. 1). In the case of the PTH1R, β-arrestin-mediated internalization does not necessarily induce G protein dissociation and termination of signaling, but can result in the formation of a stable PTH1R- β-arrestin-G protein complex that maintains G protein signaling from the endosome^17,18^. PTH binding to the PTH1R is bimodal with the N-terminal fragment (residues 1–14) of the peptide binding to the transmembrane domain and occupying the orthosteric pocket, and the C-terminal part (residues 15–34) binding to an elongated hydrophobic groove on the extracellular domain of the receptor (Fig. 1)^19^. Thus, the N-terminal fragment of the peptide represents the minimal motif needed for receptor activation^20^. Modifications of PTH by truncating the N- or C-termini or by introducing limited amino acid changes has been demonstrated to bias signaling of the receptor. In the case of PTH1R, G_s_ and G_q/11_ biased ligands with C-terminal or N-terminal truncations, respectively, have been described^21,22^. Modifications of the bovine PTH homologue led to the discovery of a β-arrestin-biased PTH peptide^23^ (Fig. 1). The concept of ligand bias has great therapeutic potential, providing opportunities to fine-tune the desired signaling outcome. Here, we aimed to discover monoclonal antibodies, with the ability to functionally modify PTH1R, using phage display. Given the importance of the ECD of the receptor for ligand binding and signaling bias, we used the isolated ECD for phage panning and screened the resulting antibodies for their ability to modulate PTH1R signaling. We identified ECD-scFvhFc, a potent single chain Fv with human Fc fragment, that acts as a β-arrestin 2 antagonist while allowing canonical G protein signaling thereby representing a valuable tool to further characterize PTH1R signaling bias.

## Results

### Isolated PTH1R ECD is pure and functional

A PTH1R ECD-Fc fusion was expressed in Expi293 cells followed by isolation using MabSelect SuRE affinity purification and subsequent removal of the Fc tag. The resulting, purified PTH1R ECD was highly homogeneous as assessed by SDS-PAGE and Western blotting analysis (Supplementary Information, Supplementary Figure 1). ITC analysis indicated that the purified PTH1R ECD bound to PTH (1–34) with a K_D_ of 4 µM when PTH1R ECD was titrated into PTH (1–34) and a K_D_ of 3.8 µM when PTH (1–34) was titrated into PTH1R ECD (Supplementary Figure 1). These values are in close agreement with the published K_D_ value of 1 µM obtained for binding of PTH (15–34) to isolated PTH1R ECD, tagged with an N-terminal MBP and a C-terminal hexa-histidine tag^24^.

### Screening for PTH1R binders

After three rounds of panning, a total of 411 phage particles specific to the PTH1R ECD, as assessed by ELISA, were isolated (Supplementary Figure 2). DNA sequencing of each resulted in 154 sequences unique at the VH CDR3 region. Those clones with identical VH CDR3 regions but different VL sequences were also taken forward as unique. scFvmFc TAP fragments of each of the 154 clones were expressed in Expi293 cells, and the culture supernatant containing the expressed scFvmFc used to confirm PTH1R binding on the cell surface. High throughput flow cytometry (HTFC) revealed that all but 33 of the 154 clones bound to the cell surface expressed PTH1R (Supplementary Figure 2). Based on cell-surface binding capability and sequence diversity, a selection of 18 scFvmFcs were selected for further analysis. To aid purification, the selected scFvmFcs were subsequently converted to scFvhFc (single chain Fv with human Fc fragment) by sub-cloning into a plasmid containing sequence which encoded a C-terminal human Fc fragment. The single-step affinity-purified samples were further checked on SDS-PAGE to ensure purity (Supplementary Figure 3) The ability of the purified antibodies to bind to cell surface expressed PTH1R was confirmed using flow cytometry (Supplementary Figure 4).

### ECD-scFvhFc has low nanomolar affinity for soluble PTH1R ECD

**Figure 2:**
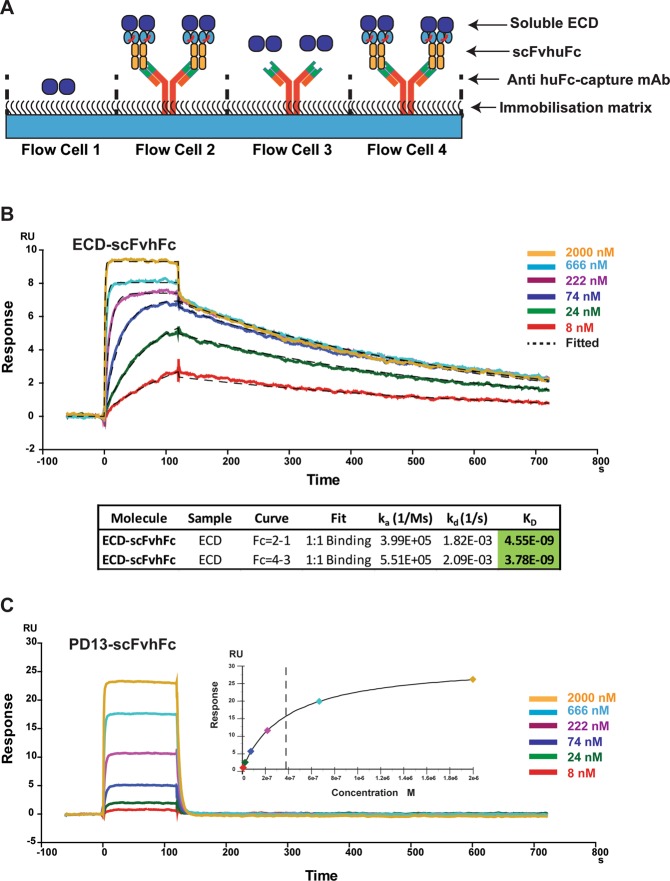
Assessment of affinity of binding of the scFv-hFcs to PTH1R-ECD by SPR. (**A**) The flow-cell capture strategy was designed to detect any non-specific binding exhibited by soluble ECD. Flow cell 1 was blocked, flow cell 3 was used to capture anti-human Fc antibody but not capture scFvhFc. Flow cells 2 and 4 were used to capture both the capture antibody and scFvhFc. Representative sensorgrams for high (**B**, ECD-scFvhFc) and low (**C**, PD13) affinity scFvhFcs are also shown. For ECD-scFvhFc, at high concentrations of ECD (>500 nM) we observed a secondary interaction characterised by fast on and off rates. The response generated to each of the ECD concentrations is indicated on the two sensorgrams. The fit of data (**C**, inset) highlights the determination of K_D_ for PD13 scFvhFc.

The purified scFvhFcs were individually captured on an SPR chip using an anti-human Fc antibody immobilized by amine coupling (Fig. 2A). The majority of the selected scFvhFc bound to the soluble ECD, with affinities in the low nanomolar (ECD-scFvhFc) to micromolar range (Supplementary Table 1). It should also be noted that the affinity data from the two different sets of flow-cells for each scFvhFc closely match, indicating that the ECD shows no non-specific binding to the immobilization matrix or to the capture antibody. Among the binders tested, ECD-scFvhFc exhibited the highest affinity for the soluble ECD with an estimated K_D_ of 4 nM and was therefore chosen for further functional analysis.

### ECD-scFvhFc is a weak cAMP antagonist

**Figure 3:**
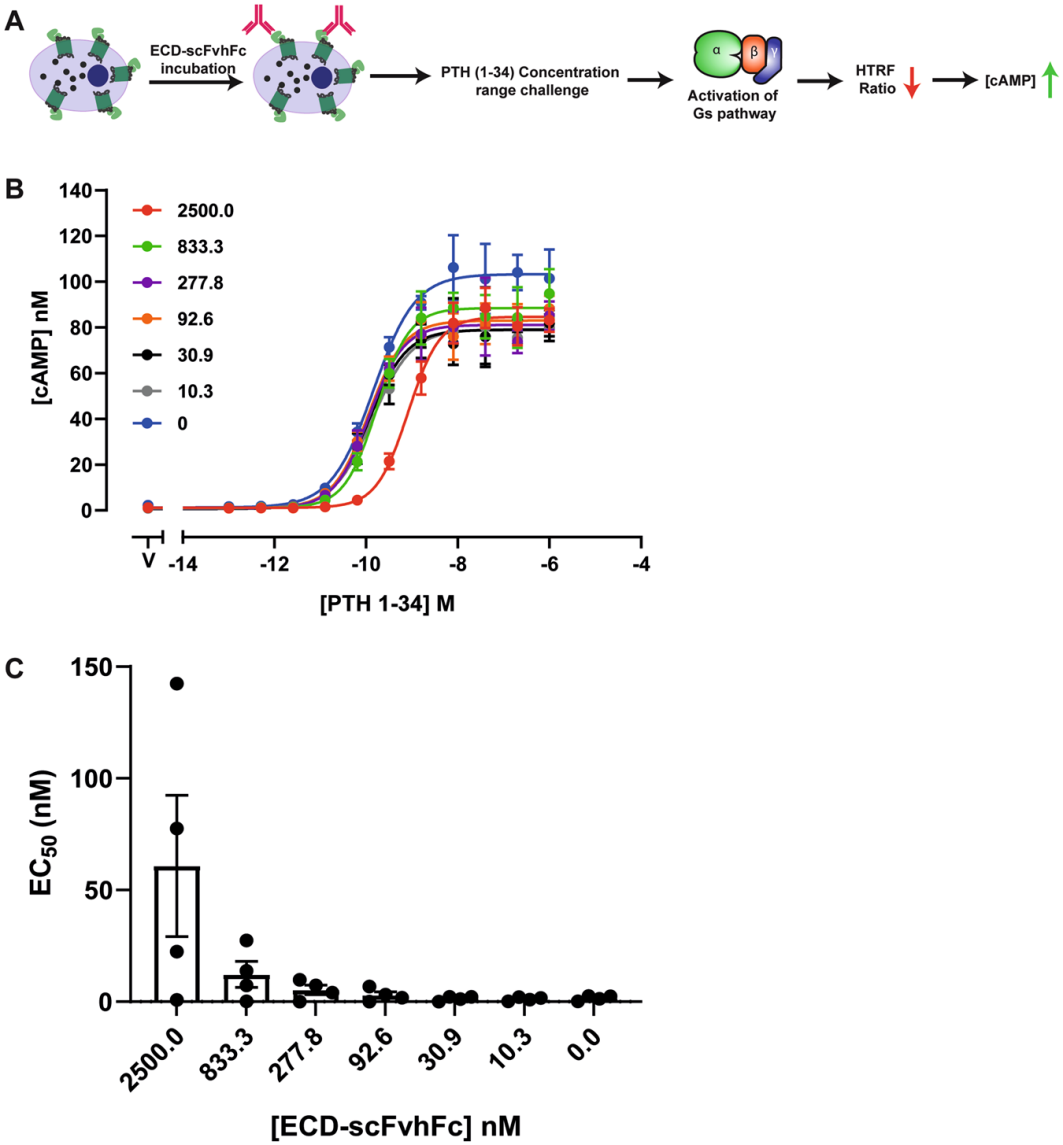
cAMP antagonism by ECD-scFvhFc. (**A**) A schematic representation outlining the effect of ECD-scFvhFc on the EC_50_ of PTH (1–34) mediated cAMP production. (**B**) A representative plot showing the effect of ECD-scFvhFc on the PTH (1–34) concentration-response curves. PTH1R expressing CHOK-1 cells were incubated with different concentrations of ECD-scFvhFc (indicated by the different coloured traces) followed by incubation with a concentration-range of PTH (1–34). The cAMP concentration was calculated using cAMP standard curve and each data point represents mean and standard deviation from quadruplicate measurements. (**C**) Plot summarising the calculated EC_50_ from four separate experiments. Bars represent means ± SEM.

The effects of ECD-scFvhFc on PTH (1–34)-induced cAMP production was assessed using an assay based on immunocompetition, where an increase in intracellular cAMP causes a decrease in the homogenous time-resolved fluorescence (HTRF) ratio (Fig. 3A). The results indicated that ECD-scFvhFc has almost no effect on the EC_50_ values of PTH (1–34) concentration response curves, with only a minor reduction in activity detected only at the highest antibody concentration (2500 nM) (Fig. 3B,C).

ECD-scFvhFc exhibited a weak effect on G_s_-mediated cAMP production when challenged with 0.4 nM of PTH (1–34) (see Supplementary Figure 5). The parental cell control showed no change in cAMP levels when challenged with either PTH (1–34) or ECD-scFvhFc (see Supplementary Figure 5).

### ECD-scFvhFc is a β-arrestin2 antagonist

**Figure 4:**
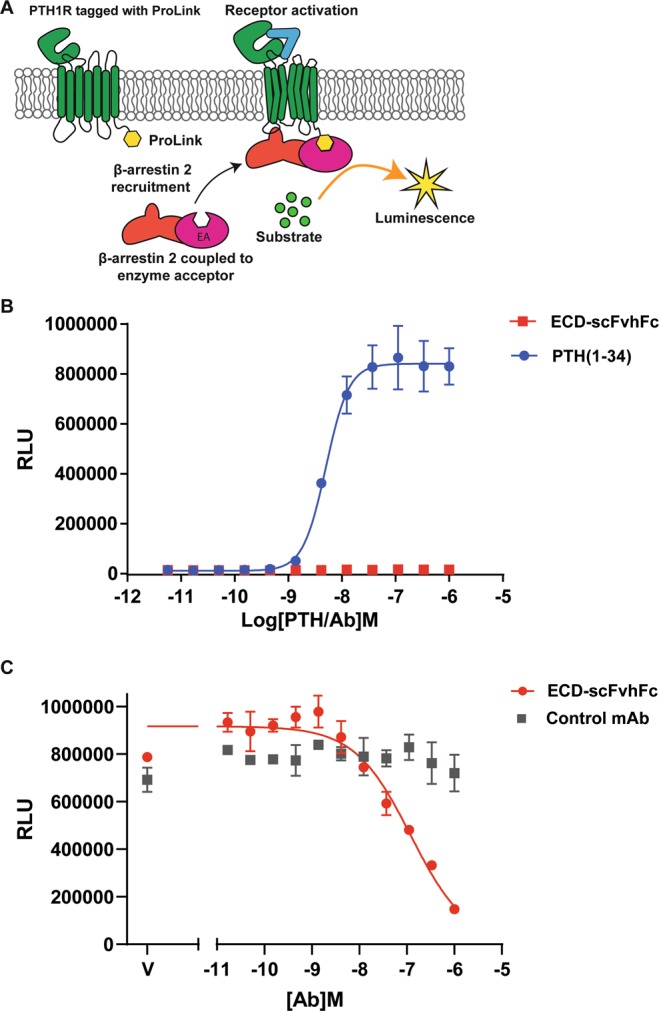
β-arrestin recruitment agonism and antagonism by ECD-scFvhFc. (**A**) Schematic representation of the β-arrestin2 recruitment assay (DiscoveRx) (**B**) PTH (1–34) (blue) and ECD-scFvhFc (red) concentration-response curve for β-arrestin 2 recruitment agonism gave an EC_50_ of 5 nM for PTH (1–34) while no agonism was seen for the ECD-scFvhFc. (**C**) Effect of ECD-scFvhFc on PTH (1–34)-mediated β-arrestin2 recruitment shows a concentration dependent antagonism (red), while control mAb (gray) exhibited no response. Representative data from three biological replicates where each datapoint represents duplicate measurements.

The effects of ECD-scFvhFc on PTH1R coupling to β-arrestin 2 were assessed using an enzyme fragment complementation assay. The receptor-β-arrestin 2 complex forms a fully functional β-galactosidase which produces a luminescent product when substrate is added (Fig. 4A). ECD-scFvhFc was tested in the agonist format of the assay along with PTH (1–34). PTH (1–34) induced receptor coupling to the β-arrestin 2 in a concentration dependent manner with a pEC_50_ = −8.30 ± 0.05 (in line with the values reported by the manufacturer), whereas no β-arrestin 2 recruitment occurred when ECD-scFvhFc was added alone (Fig. 4B). However, ECD-scFvhFc antagonizes PTH (1–34) induced β-arrestin 2 recruitment (Fig. 4C). In these experiments PTH (1–34) was used at a concentration of 10 nM. The calculated IC_50_ value for antagonism by ECD-scFvhFc was found to be 102.3 ± 14.8 nM (mean ± SEM from three separate experiments performed in duplicate with the raw RLU fitted by constraining the lowest value equal to 0).

### ECD-scFvhFc binds the full-length receptor with high affinity

**Figure 5:**
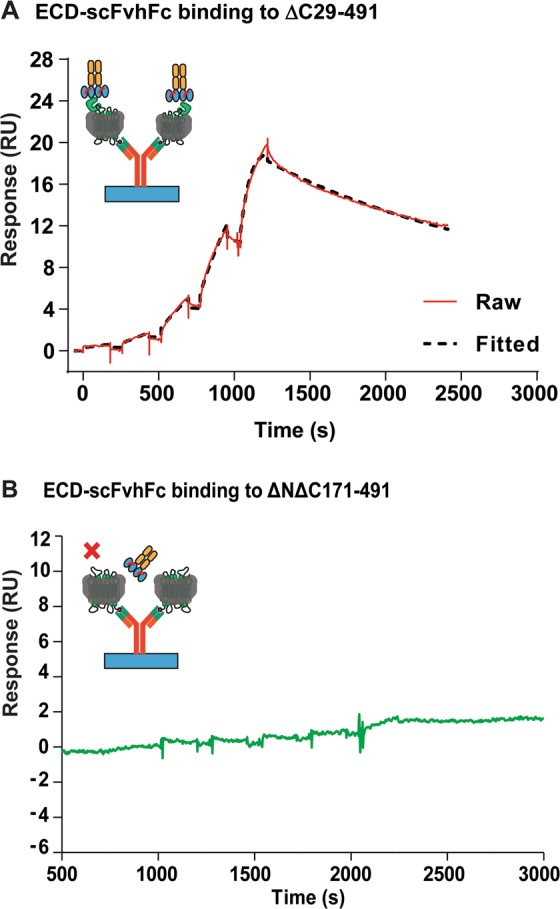
Kinetics analysis of ECD-scFvhFc binding to purified PTH1R receptor constructs. Single kinetics analysis of ECD-scFvhFc binding to the SMA-solubilized (**A**) ΔC29-491 and (**B**) ΔNΔC171-491 constructs. While the mAb exhibits concentration-dependent binding to the ΔC29-491 construct, in the absence of ECD, the scFvhFc shows no binding to the ΔNΔC171-491 construct (**B**).

His-tagged receptor variants enriched using affinity capture as styrene maleic co-polymer lipid particles (SMALPs) were used to determine the binding kinetics of ECD-scFvhFc by surface plasmon resonance (Biacore T200). The receptor variants (ΔC29-491 and the ΔNΔC171-491, i.e. with or without the N-terminal ECD) were immobilized on to the Biacore chip with a His-tag specific polyclonal antibody. ECD-scFvhFc bound to the ΔC29-491 construct with a K_D_ ~4 nM (Fig. 5A and Table 1). The ScFvhFc was not observed to bind to the ΔNΔC171-491 construct which lacks the ECD (Fig. 5B and Table 1).

**Table 1:** Kinetic data for ECD-scFvhFc binding to SMA-stabilized PTH1R constructs. Due to the limitation of mAb the highest concentration used in the experiment was 2 μM and KD was estimated under no saturation conditions.

Run-1					
Sample^1^	Construct^2^	k_a_ (1/Ms)^3^	k_d_ (1/s)^4^	K_D_ (M)^5^	t_1/2_ (min)^6^
ECD-scFvhFc	∆C29-491	8.78E + 04	2.81E-04	**3.20E-09**	40.93
ECD-scFvhFc	∆N∆C171-491	N/A	N/A	**N/A**	**N/A**
**Run-2**					
**Sample**	**Construct**	**k**_**a**_ **(1/Ms)**	**k**_**d**_ **(1/s)**	**K**_**D**_ **(M)**	**t**_**1/2**_ **(min)**
ECD-scFvhFc	∆C29-491	1.13E + 05	4.54E-04	**4.04E-09**	25.33
ECD-scFvhFc	∆N∆C171-491	N/A	N/A	**NA**	**N/A**

^1^Antibody details.

^2^SMA-stabilised PTH1R construct immobilised and used for mAb binding.

^3^k_a_ = on-rate.

^4^k_d_ = off-rate.

^5^K_D_ represents the measured affinity in M.

^6^t_1/2_ represents the complex half-life values, (t_1/2_ = ln(2)/k_off_).

### ECD-scFvhFc binds to the N-terminal region of PTH1R ECD

**Figure 6:**
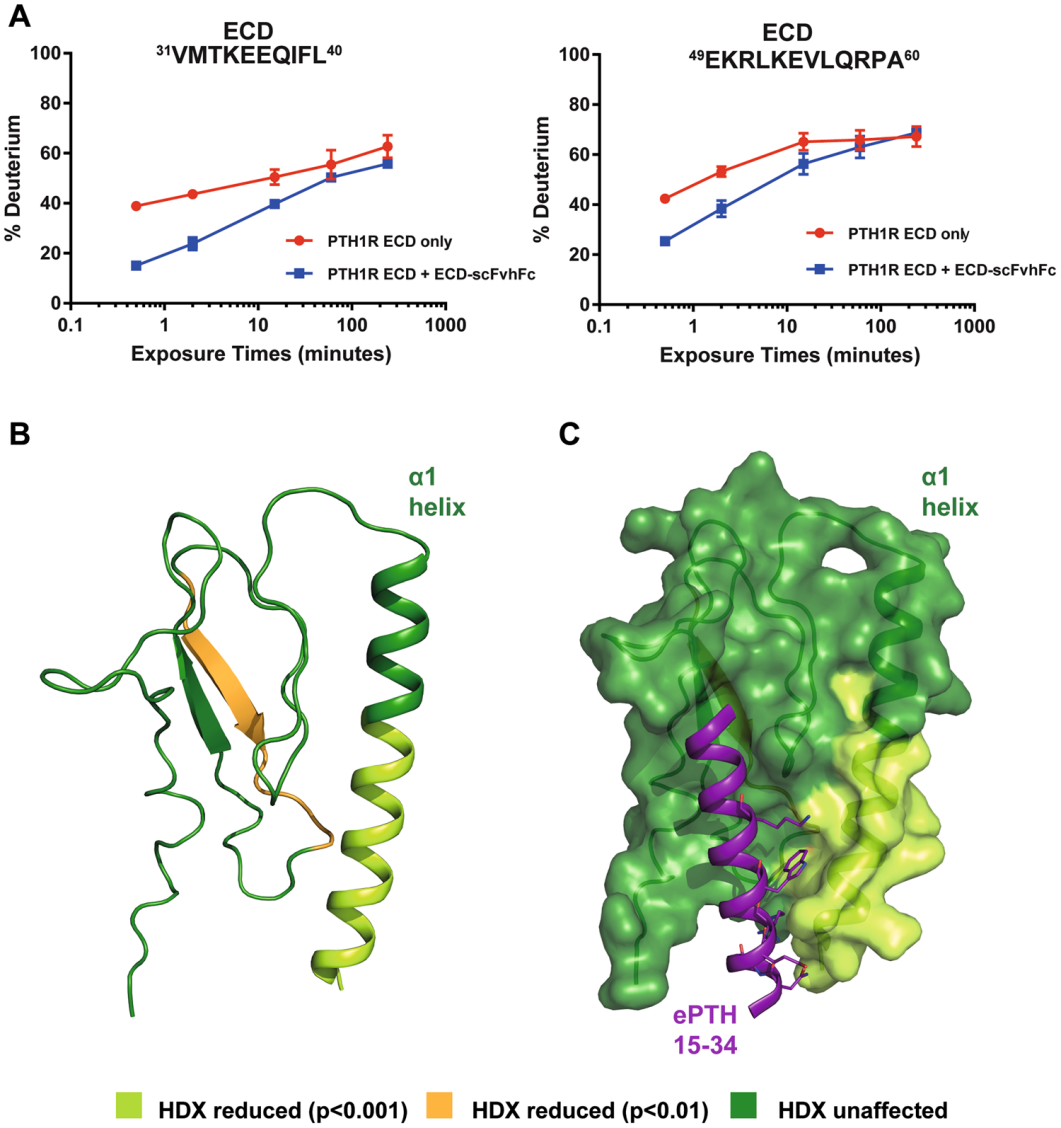
HDX-MS analysis of PTH1R ECD bound to ECD- scFvhFc. (**A**) Deuterium uptake plot showing the difference in apo (red) and ECD-scFvhFc bound PTH1R-ECD (blue). Peptides corresponding to the sequence ^31^VMTKEEQ----EVLQRPA^60^ exhibited slower exchange rates when bound to ECD-scFvhFc. (**B**) The putative epitope of ECD-scFvhFc is located on the α1-helix on the PTH1R-ECD and is highlighted in light green. The additional peptide showing slower HDX is depicted in orange, areas of the PTH1R-ECD that showed no change in HDX are coloured dark green. (**C**), Surface representation of the PTH (15–34)-bound PTH1R-ECD reveals an overlap of the putative ECD-scFvhFc epitope and the PTH (15–34) binding site. Residues interacting with the PTH1R-ECD in that area are highlighted as sticks. The second peptide that showed reduced HDX (orange) is located opposite the PTH (15–34) binding site and does not overlap. The figure was prepared using PDB 6FJ3^19^.

Hydrogen-deuterium exchange mass spectrometry (HDX-MS) uses the rate of exchange of protein backbone amide hydrogens with deuterium ions in bulk solution as a measure of structural dynamics. Areas of intermolecular interaction are more protected against this exchange; thus HDX-MS can be used to identify binding sites. Here, HDX for PTH1R-ECD alone and bound to ECD-scFvhFc was compared. After labelling, the protein was digested with pepsin into peptides, and the difference in deuterium incorporation calculated per peptide. Peptides were identified covering 99% of the ECD sequence. Overlapping peptides assigned to the N-terminus of the ECD, covering the sequence ^30^DVMTKEEGIFLLHRAQA^46^, were found to be more protected from exchange in the ECD-scFvhFc complex (Fig. 6A and Supplementary Table 2), ΔHDX% apo versus bound p < 0.001)). Two further regions also showed lower uptake of deuterium in the presence of the scFvhFc: ^62^IM^63^ (these residues are not resolved in available crystal structures) and ^125^EVVAVPCPDY^134^ (ΔHDX% apo versus bound p < 0.01). It should be noted that since the results reflect peptide-level resolution, it is not possible to discern which of the residues within these regions are most important for the ECD-scFvhFc interaction, and protection may be due directly to binding or as an indirect effect e.g. the stabilisation of adjacent structural elements. Mapping the HDX-MS results onto the crystal structure of PTH1R-ECD (PDB ID: 3C4M, Fig. 6B) reveals that the affected peptides cluster close to the PTH (1–34) binding site. Indeed, the region most affected by scFvhFc binding is the α1 helix, some residues of which are known to interact directly with PTH (1–34) (Fig. 6A,B). However, other residues known to be involved in the ECD-PTH (1–34) interaction are not identified as being protected here. This suggests a partial overlap of the ECD-scFvhFc epitope with the known PTH (1–34) binding site.

## Discussion

The importance of the ECD for ligand binding and receptor activation has been described for several secretin class receptors^25^ and this prompted us to raise antibodies against this domain to functionally modify the receptor. In this study, we have shown that ECD-scFvhFc binds the PTH1R with high affinity while allowing PTH (1–34) to bind and exert its agonistic signaling properties. When bound to the PTH1R, ECD-scFvhFc effectively blocks β-arrestin 2 coupling, thus stabilizing the receptor in an active, but β-arrestin 2-antagonistic conformation.

ECD-scFvhFc was discovered against the ECD of the receptor and binds to the isolated ECD with high affinity (4.6 nM, Fig. 2, Supplementary Table 1). When assessing the affinity of ECD-scFvhFc for the full-length PTH1R we used SMALP isolated receptor and found that the affinity of ECD-scFvhFc for both the isolated ECD and the full-length receptor is comparable (~4 nM in each case, Fig. 2 and Fig. 5) confirming that raising antibodies against the isolated ECD of secretin-family receptors can result in high affinity receptor binders. SMALPs provide several advantages over classical membrane protein preparations as they directly extract the target protein from the membrane without the need to expose the protein to detergents^26^. In our hands, although the purity of the receptor preparation in SMALPs was limited, the SMALP-solubilized protein was stable on an SPR chip. This allowed characterization of the binding kinetics of ECD-scFvhFc in the absence of detergents which have been shown to influence the binding of antibodies to membrane proteins^27^. SMALPs are reported to retain endogenously bound lipids which may be crucial for the correct folding and function of a membrane protein and therefore represent a promising technology to study membrane proteins in purified form while keeping their environment as native as possible. Several lipids were resolved in the recently determined crystal structure of the PTH1R^19^ which may be relevant for receptor stability and functionality, underlining that retaining endogenously-bound lipids by using SMALPs could be a suitable format for studying PTH1R function.

**Figure 7:**
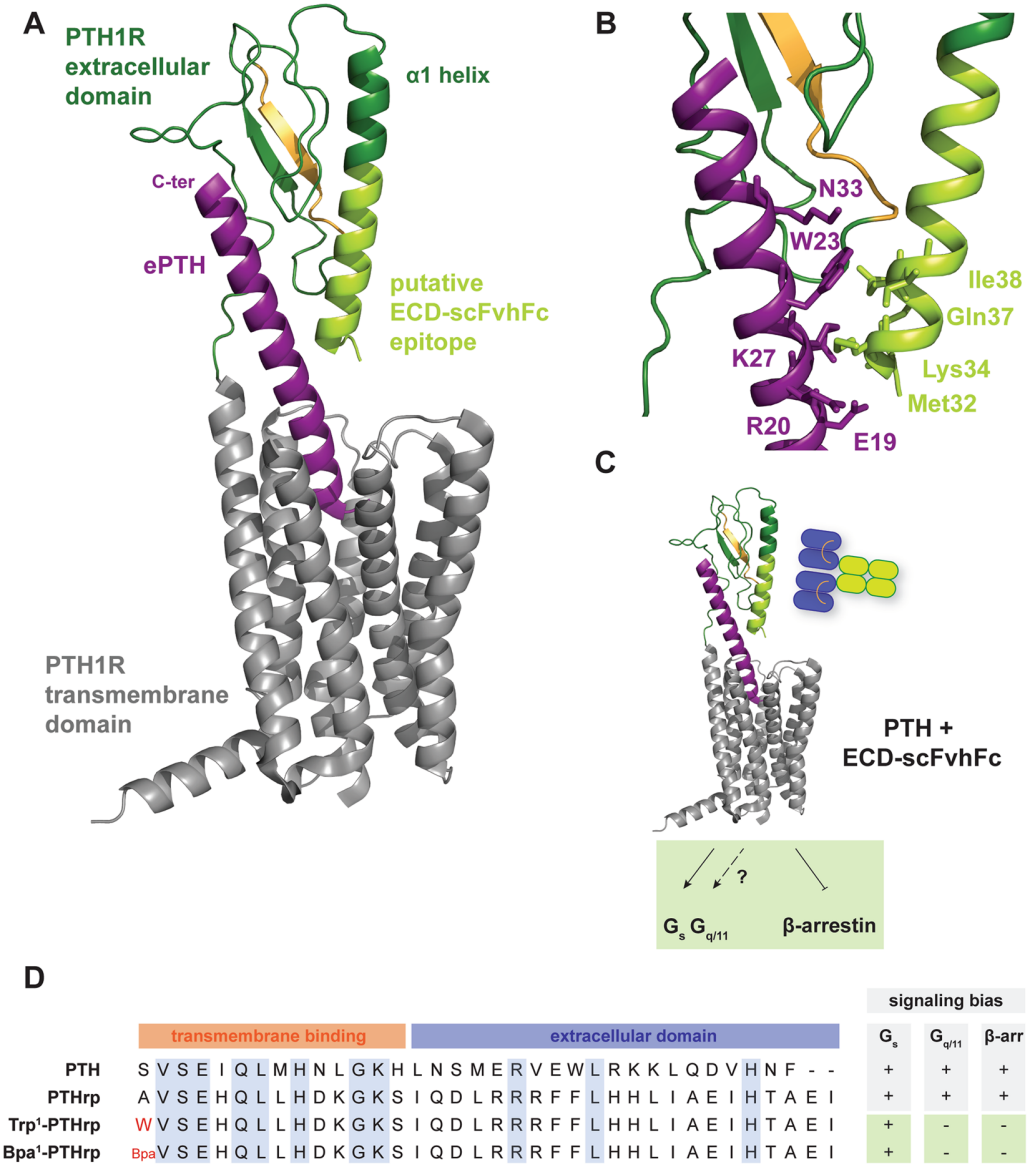
Visualization of the predicted epitope of ECD- scFvhFc on the PTH1R-ECD and the sequence diversity of β-arrestin-biased PTH variants highlights the complexity of PTH signaling. (**A**) The epitope of ECD- scFvhFc was determined using HDX-MS and is located in the α1 helix of the PTH1R-ECD (highlighted in light green). (**B**) Residues in the PTH1R-ECD interacting with ePTH (an engineered PTH used for structure determination (Ehrenmann *et al*., 2018, PDB 6FJ3) and overlapping with the putative epitope of ECD- scFvhFc are shown as light green sticks and numbered in three letter amino acid code (light green). Amino acids in ePTH interacting with these residues are shown as purple sticks and labeled using single letter amino acid code (purple). (**C**) Summary of the signaling behavior of PTH in the presence of ECD-scFvhFc. (**D**) Alignments of PTH, PTH related peptide (PTHrP) and β-arrestin-antagonizing variants. Abbreviations: Bpa, p-benzoylphenylalanine.

The epitope of ECD-scFvhFc was suggested by HDX-MS to be located on and perhaps adjacent to the α1 helix of the ECD (Fig. 6). Some residues on this helix interact with the central part of PTH (16–27), however these residues may not be directly involved in the scFvhFc binding. Although, ECD-scFvhFc was raised against the apo ECD, i.e. without PTH present, the antibody does not appear to directly compete with PTH binding since it had little effect on PTH (1–34) mediated G_s_ activation (Supplementary Figure 5). ECD-scFvhFc also has no effect on the EC_50_ of PTH (1–34)-induced cAMP production and only marginally shifts the EC_50_ at very high concentrations of antibody used in the assay (2500 nM, Fig. 3A,B) emphasizing that ECD-scFvhFc does not compete directly with PTH (1–34) at physiological concentrations. In addition, ECD-scFvhFc alone cannot act as a PTH1R agonist (data not shown). This was expected given that binding of the N-terminal region of PTH to the orthosteric site in the transmembrane domain of PTH1R is essential for G protein activation (Fig. 1)^20^. In contrast, ECD-scFvhFc is a potent inhibitor of PTH1R β-arrestin 2 recruitment (IC_50_ 102.3 ± 14.8 nM) and thus may extend PTH-induced G protein signaling. ECD-scFvhFc appears to be a β-arrestin 2 antagonist while allowing PTH to initiate receptor signaling through G_s_. The only ligands described to date that possess the same signaling behavior on the PTH1R are variants of PTH related peptide (PTHrP)^21,22,28,29^, (Fig. 7). PTHrP and derivatives share high sequence homology in the N-terminal (i.e. orthosteric) fragment with PTH but the C-terminal region is diverse. In contrast to PTH, PTHrP has been described to mainly play a role in endochronal bone development and other endocrine developmental processes^30–32^. The ECD-binding fragments (15–34) of PTHrP and PTH both bind to the same hydrophobic groove on the surface of the ECD with residues 15–24 displaying similar conformations and interactions with the receptor whereas the C-terminal region of PTHrP undergoes partial unwinding of the helix and bends away from the α1-helix of the ECD^33^. Given that altering the N-terminal residues of PTHrP results in peptides that are selective for G_s_ but demonstrate β-arrestin antagonism,^21,22,28,29^ it is tempting to speculate that ECD-scFvhFc distorts the conformation of the ECD to convey signaling properties to PTH that are comparable to Trp^1^-PTHrP and Bpa^1^-PTHrP (Fig. 7C,D). In addition to being β-arrestin antagonists, Trp^1^-PTHrP and Bpa^1^-PTHrP are also antagonizing G_q/11_ signaling which has not been assessed in this study and could add to the complete characterization of ECD-scFvhFc’s signaling behavior.

The detailed characterization of PTH, PTHrP and their engineered variants has led to a good understanding of the determinants of PTHR1 signaling behavior. PTH signaling eventually results in osteoblast stimulation, bone mineralization and bone formation but also triggers bone resorption via the indirect activation of the RANKL and OPG pathways^34^. β-arrestin recruitment to the receptor is thus needed to terminate G protein signaling and maintain a balance between bone formation and resorption.^16^ Particularly when exploiting the PTH1R-PTH signaling axis for therapeutic purposes, a balanced effect of the ligand of choice is desired and the potential of biased GPCR ligands as drugs has been widely discussed.^35^ In the case of PTH1R, achieving such a balance of signaling properties has proven difficult when attempting to modulate the receptor using small molecule ligands. AH-3960^36^ and SW106^37^ were some of the first PTH1R small molecule ligands described and act as agonists of the receptor.^38^ These ligands act exclusively by occupying the orthosteric pocket of the receptor and do not require the presence of the ECD to activate the receptor.^38^ In an attempt to treat hypoparathyroidism, the small molecule agonist PCO371 which also mainly acts via the orthosteric pocket in the transmembrane bundle, was developed.^39^ Although these molecules contribute largely to the understanding of PTH1R binding and signaling, they suffer from low affinity and poor drug-like properties.^38,39^ Given the complex signaling behavior of PTH and PTH variants as well as the receptor’s bimodal binding mode requiring the ECD for high affinity interaction, it is not surprising that it has proven difficult to identify potent small molecule ligands with the desired signaling profile. Up to now, the only approved drug to treat bone loss is recombinant PTH, teriparatide, which is used intermittently to favor bone formation over bone loss.^40^ Given the difficulties in developing drugs that modify PTH1R function, attempting to modulate receptor function using a therapeutic antibody represents a promising alternative. Therapeutic monoclonal antibodies against several classes of GPCRs have been described including secretin-class GPCRs,^41^ one example being AMG 334 (Erenumab) targeting the Calcitonin Gene-Related Peptide Receptor^42^ which has now reached the market and is used in migraine prevention. A PTH1R antagonistic antibody has been described, which was identified by cell-based phage panning against the full-length receptor (US patent 2018/0030154A1) but it is not clear whether this antibody is a direct competitor of PTH binding and inhibits PTH1R signaling by blocking access of the agonist to the receptor binding site. Although the mechanism of ECD-scFvhFc-mediated PTH1R modulation is not fully understood and the signaling efficacy of ECD-scFvhFc is not directly therapeutically relevant, this study underlines the potential of antibodies to modulate GPCR signaling and further use of this antibody may help to decipher the complex signaling bias of the PTH1R.

## Materials and Methods

### Purification and characterization of the PTH1R ECD

PTH1R ECD-TEV-human Fc (residues M1-G188, Uniprot: Q03431) was expressed using the pMH vector (UCB proprietary) using the Expi293 system (Thermo Fisher). The ECD was captured from the supernatant using Protein A affinity resin (MabSelect SuRE, GE Healthcare) in batch. The resin was washed with 20 CVs of Tris-buffered saline (TBS) and incubated overnight with 200 µg of TEV-6His protease, at 4 °C. The resin was sedimented and the supernatant containing TEV protease and PTH1R-ECD was recovered. Ni-NTA beads were used to remove TEV-6His and the PTH1R- ECD was buffer exchanged into TBS pH 8.0 using 50 kDa molecular weight cut-off filters (Amicon, Millipore), concentrated to 11 mg/ml, flash-frozen and stored at −80 °C for future use. The protein was assessed for purity by SDS-PAGE and Western blot analysis. PTH (1–34) binding function of the purified ECD was confirmed using isothermal titration calorimetry (ITC).

### Generation and characterization of anti-PTH1R ECD mAb

PTH1R ECD-specific antibodies were generated by phage display using the isolated PTH1R ECD protein as target. An in-house (UCB Pharma SA, unpublished) human naïve scFv phage library, with a diversity of approximately 1 × 10^11^ colony forming units (cfu), was used for phage display panning on the recombinant protein. PTH1R-ECD (10 µg/ml, 50 µl) was immobilized on plastic (Maxisorp, Nunc) overnight at 4 °C. Plates were washed with PBS / 0.1% Tween 20 and blocked for 1 h at room temperature using (PBS supplemented with 3% BSA, 300 µl). Phage particles were blocked for 1 h at room temperature (3% BSA, 3% fat-free milk in PBS), plates were washed and 50 µl of phage particles were added to the ECD coated wells and incubated at room temperature for 1 h. The plates were washed five times to remove unbound phage particles and the antigen binding phage were eluted with 0.1 M HCl, neutralized using 0.1 M Tris, pH 7.4 and used to infect TG-1 cells (Lucigen Technologies) at an OD_600_ of between 0.5–0.8 and plated on Luria Bertani (LB) agar supplemented with 100 µg/ml carbenicillin and 1% glucose (LBAG) prior to incubation overnight at 37 °C. Phage rescue was performed as described in supplementary methods. Subsequent rounds of phage panning used a lower ECD plating concentration (1 µg/ml) and the plates were washed 20 times to increase stringency. In addition, for the third and final panning round, all bacterial culture steps were performed using XL1 Blue cells (Agilent Technologies) instead of the TG-1 cells. Single colonies were picked, rescued as described and analysed by monoclonal phage ELISA as described in supplementary methods.

### Generation and expression of the scFvFc molecules

A two-stage PCR based approach was used to convert selected scFvs to a transcriptionally active fragment containing a C-terminal murine gamma 1 Fc fragment (scFvmFc)^43^. The scFvmFc constructs were expressed in 1 ml cultures of Expi293 cells as described above for the ECD.

### Screening of scFvmFc using high-throughput flow-cytometry (HTFC)

Expi293 cells were transiently transfected with ΔN (T178-M593) and WT PTH1R (D27-M593) constructs, both flanked with N-terminal HA signal sequences, FLAG, 3 C protease sites and C-terminal Avi tag, TEV site, His-Lys-His tags. Cells were harvested after 24 hr incubation, washed twice with Flow Buffer (PBS pH 7.4, 1% BSA, 0.1% sodium azide and 2 mM EDTA) and re-suspended in Flow Buffer at a density of 1 × 10^6^ cells/ml. 5 μl of Expi293 media containing scFvmFc were added and non-specific binding was assessed using ΔN transfected cells. Plates were incubated on ice for 1 h and washed twice with Flow Buffer. Goat anti-mouse IgG Fc-fragment specific IgG coupled to Alexa Fluor 647 (1: 1000 diluted using flow buffer) was added and further incubated on ice for 1 h. Plates were washed, and cells resuspended in 50 μl of Flow Buffer and analyzed using the IntelliCyt iQue high-throughput flow cytometry (HTFC). An iQue instrument with a 2 laser (blue, red) configuration was used to perform the HTFC. The live cells were gated using a FSC-H vs. SSC- H dot plot and the data for Alexa Fluor 488 and 647 emissions were recorded using the FL1 and FL4 filters.

### Expression and purification of selected scFvhFc

A total of 18 scFvmFcs were converted to scFvhFc (scFv with human Fc) and expressed in Expi293 cells as described. scFvhFc was captured from the supernatant using a 5 ml HiTrap Protein A HP column (GE Healthcare). The column was washed with PBS followed by elution using 0.1 M Sodium citrate, pH 3.1. Elution fractions were immediately neutralized using 2 M Tris, pH 8.0 and the concentration was normalized to ~1 mg/ml (or 10 μM, assuming an average molecular weight of 100 kDa) with PBS pH 7.4.

### Flow cytometry analysis

Flow cytometry analysis was essentially performed as described for HTFC above with the following changes: ~10^5^ cells were added to each well of a 96 well U-bottom plate and 5 μM purified scFvhFc. The plates were incubated on ice for 1 h and washed twice with Flow Buffer. Goat anti-human IgG Fc- Alexa Fluor 488 secondary antibody (1: 10 diluted in flow-buffer) was added to each well and the plates incubated for a further 1 h on ice in the dark. The plates were washed twice, and the cells re-suspended in 200 μl flow-buffer and analyzed a BD FACS Canto HTS equipped with the 3-laser configuration was used to perform the flow- cytometry. A total of 10^4^ events of the singlet gate were used for the data acquisition, with the flow-rate set to medium. Data were analysed in FlowJo V10 software (FlowJo LLC). The geometric mean of three technical replicates of the 488-nm fluorescence was plotted using GraphPad Prism 7.0.

### Kinetics of scFvhFc binding using Surface Plasmon Resonance (SPR)

A Biacore T200 (GE Healthcare) was used to perform the SPR, to measure the binding kinetics of the scFvhFc binding to the soluble ECD. Using amine coupling, a goat anti-human Fc capture antibody (Jackson Immunoresearch) was immobilized on a CM5 chip to give a final capture level of 600–900 response units (RU). The scFvhFc molecules were diluted to 2–3 μg/ml using the ECD running buffer (1x HBS-EP + supplemented with 350 mM NaCl) to give final capture levels of ~100 RU. Starting at 2 μM the ECD was flowed over using a 3-fold dilution series and the binding kinetics were determined using the multi-cycle approach. The data analysis was performed using the Biacore T200 evaluation software and a 1:1 Langmuir binding model as used to fit the binding curves, unless otherwise stated.

### Effect of ECD-scFvhFc on cAMP signaling

The effect of ECD-scFvhFc binding on G_s_-mediated cAMP signaling was probed by investigating its effect on PTH (1–34) concentration-response curves. Typically, 3000 CHOK-1 cells stably expressing PTH1R (DiscoveRx) (in PBS + 0.5 mM IBMX) were incubated with different concentrations of ECD-scFvhFc (2500 nM to 10 nM) for 30 minutes at room temperature. Following incubation, a concentration range (5-fold serial dilution from a starting concentration of 1 μM) of PTH (1–34) was added followed by 30 minutes incubation at 37 °C. The intracellular cAMP levels were detected using the cAMP dynamic 2 kit following manufacturer’s protocol. Briefly, 5 μl each of cAMP-d2 and Anti-cAMP-cryptate, diluted in 1x lysis and detection buffer, were added to each well followed by an hour incubation at room temperature in the dark. A HTRF-enabled plate reader was used to determine the ratio of absorbance at 665 to 620 nm. A cAMP standard curve was generated in similar manner to allow calculation of final cAMP concentration in each well. The cAMP concentration (in nM) and the standard deviation was plotted using GraphPad Prism and fitted using four-parameter logistic regression. Each data point represents quadruplicate measurement with four biological replicates. The cAMP dynamic 2 kit (Cisbio) was also used to determine the functional effects of the ECD-scFvhFc. In agonist mode, cells were treated with increasing concentrations of PTH (1–34) or ECD-scFvhFc. For determining antagonism, cells were incubated with a concentration range of ECD-scFvhFc for 30 min at room-temperature followed by a PTH (1–34) challenge at 0.4 nM concentration. The cAMP concentration and standard deviation for each data point were plotted using GraphPad Prism 7.

### Analysis of β-arrestin 2 coupling

The β-arrestin 2 recruitment assay was performed using a PathHunter β-arrestin eXpress kit based on enzyme fragment complementation (EFC) technology. 10^4^ cells were plated in a white 96-well plate (DiscoveRx) and incubated for 48 hr in a 37 °C incubator with 5% CO_2_ and in a humidified environment. Following incubation, the cells were used for assay in either agonist or antagonist mode. In agonist mode, increasing concentrations of PTH (1–34) and ECD-scFvhFc were added and incubated for 90 mins in a 37 °C incubator. In antagonist mode, cells were incubated with increasing concentrations of ECD-scFvhFc for 30 min at 37 °C and then challenged with PTH (1–34) at EC_80_ concentration and substrates for detection were added as described by the manufacturer. The final luminescence from each well was read using a Synergy Neo 2 plate reader. Data were plotted and fitted with a four-parameter logistic regression using GraphPad Prism 7 and are represented as means ± SD. The final IC_50_ value was calculated from three separate experiments performed in duplicate.

### SMA-solubilization and purification of PTH1R constructs

C-terminally truncated [amino acids D29 to S491(ΔC29-491)] and N and C-terminally truncated [amino acids V171 – S491 (ΔNΔC171-491)] constructs of PTH1R incorporating an N-terminal twin Strep II tag and a C-terminal deca-histidine tag were expressed in Expi293 cells as described. Typically, 0.8–1 gram of membrane from PTH1R-construct expressing Expi293 cells were re-suspended in SMA solubilization solution (50 mM Tris, pH 8.0, 150 mM NaCl, 10% glycerol, and 2.5% w/v SMA polymer) to give a final membrane concentration of 30–40 mg/ml (by weight). The suspension was incubated for 1–2 h at room-temperature with slow-shaking. For these experiments two sources of SMA polymer were used: UCB-SMA, sourced from Cray Valley UK and hydrolyzed at UCB following published conditions^44^ and pre-hydrolyzed SMA from University of Birmingham (kind gift from Prof. Mark Wheatley). Insoluble material was removed by centrifuging the suspension at 190,000 x g for 1 h at 4 °C, and the supernatant containing the solubilized receptor was incubated with 1 ml of Ni^2+^-NTA resin (Qiagen) and incubated at 4 °C overnight with gentle shaking. All further purification steps for the SMA solubilized receptor were performed at room temperature. After overnight incubation, the Ni^2+^-NTA beads were collected and washed with 20 CVs of MSB (50 mM Tris pH 8.0, 150 mM NaCl, 10% glycerol) and bound receptor was eluted with MSB supplemented with 10, 20, 40, 80 or 500 mM imidazole. The relevant fractions were pooled and concentrated using centrifugal-filtration devices with a 100 kDa MWCO filter (Millipore).

### Surface plasmon resonance (SPR) analysis of ECD-scFvFc binding to purified PTH1R

SPR measurements were used to determine the affinity of ECD-scFvhFc for PTH1R constructs. Binding assays were carried out using a Biacore T200 instrument (GE Healthcare Bio-Sciences AB). Anti-His antibody (GE Healthcare Bio-Sciences AB) was diluted to 50 μg/ml in 10 mM sodium acetate, pH 4.5 buffer and immobilized on a CM5 Sensor Chip (GE Healthcare Bio-Sciences AB) via amine coupling chemistry to a level of ~5000 response units (RU). All binding assay steps were performed using a running buffer comprised of 50 mM Tris, 500 mM NaCl, 0.005% (v/v) P20, pH 8.1. Single step affinity purified ΔC29-491 and ΔNΔC171-491 constructs at concentrations of 0.2–0.3 mg/mL were applied to achieve 200–300 RU capture using the C-terminal deca-histidine tag and immobilized anti-His-tag antibody. ECD-scFvhFc was titrated over the captured ΔC29-491 or ΔNΔC171-491, respectively, at concentrations ranging from 2 to 2000 nM using single cycle kinetics. The flow rate used for the binding assay was set to 30 μL/min. The chip surface was regenerated by flowing 50 mM HCl twice at a flow-rate of 10 μL/min for 90 s each. A blank run with the same condition was performed prior to the antibody sample injection. Background subtracted binding curves were analyzed using the T200 evaluation software (version 3.0) following standard procedures and kinetic parameters determined using the 1:1 binding model.

### Analysis of ECD-scFvhFc interaction using HDX-MS

HDX-MS was used to determine the difference in deuterium uptake of the PTH1R-ECD upon ECD-scFvhFc binding. 20 μM ECD was analyzed alone or pre-incubated with 20 μM ECD-scFvhFc. 3 μl of the protein solution was mixed with 57 μl of the H_2_O equilibration buffer (time 0) or the D_2_O labelling buffer (incubated at 20 °C for 30 s, 2 min, 15 min, 1 hr or 4 hr). The equilibration and labelling buffers were 10 mM potassium phosphate in H_2_O (pH 7.0) or D_2_O (pD 7.0), respectively. 50 μl of each reaction mixture was added to 50 μl of quench solution (3.4 M guanidinium-HCl, 62.5 mM TCEP, 100 mM phosphate, pH 2.4) cooled to 0 °C, and injected either immediately or after a 2 minute ‘quench-hold’ into the Waters HDX module and Acquity M-Class UPLC system (50 μl sample loop). Upon injection, the sample was washed over a Waters Enzymate pepsin column using 0.02% HCOOH in H_2_O at a flow rate of 100 μl/min with a pressure of approximately 6500 psi. Resulting peptides were trapped on a Waters Acquity UPLC BEH C18 VanGuard trapping column (2.1 × 5 mm, 1.7 μM particle) over 3 minutes. Peptides were then washed off the trap column onto an analytical Waters Acquity UPLC BEH C18 column (100 × 1.0 mm, 1.7 μM particle), and the following gradient used for elution: 0 mins: 5% B; 6 mins: 35% B; 7 mins: 40% B; 8 mins 95% B; flow rate 40 μl/min (Solvent A = 0.02% HCOOH in H_2_O; Solvent B = 0.02% HCOOH in MeCN) followed by 2 wash steps. Mass spectrometry data were collected on a Waters Synapt G2Si instrument, over the m/z range 50–2000 Th, with a scan rate of 0.3 s. MSe data were acquired using a trap collision energy ramp of 18 to 40 V for the high-energy function. Mass calibration was performed using the MS/MS spectrum of doubly charged [Glu-1]-fibrinopeptide B, and lockmass data on the same peptide were acquired for mass correction during data processing. Data were collected in triplicate, with a blank run performed between each set of 3. Data were processed using PLGS v3.0.2 and DynamX v3.0 software packages from Waters (see Supplementary Methods for more details). The 0.5 min time point was used for statistical analysis and definition of the epitope, as it represented the largest changes in HDX dynamics between bound and unbound states. Final data were plotted using GraphPad Prism 7 representing mean and standard deviation of the triplicate measurement.

## Supplementary information

Supplementary information
