# Supplementary material for: Modulation of PTH1R signaling by an ECD binding antibody results in inhibition of β-arrestin 2 coupling
Source: Sci Rep. 2019 Oct 8;9:14432. doi: 10.1038/s41598-019-51016-z (PMC6783463; doi:10.1038/s41598-019-51016-z)
Supplement: Supplementary file 1 — Supplementary information [file 41598_2019_51016_MOESM1_ESM.docx]

# Supplementary Information

## Supplementary Figure 1: Isolation and characterization of the PTH1R ECD.

**
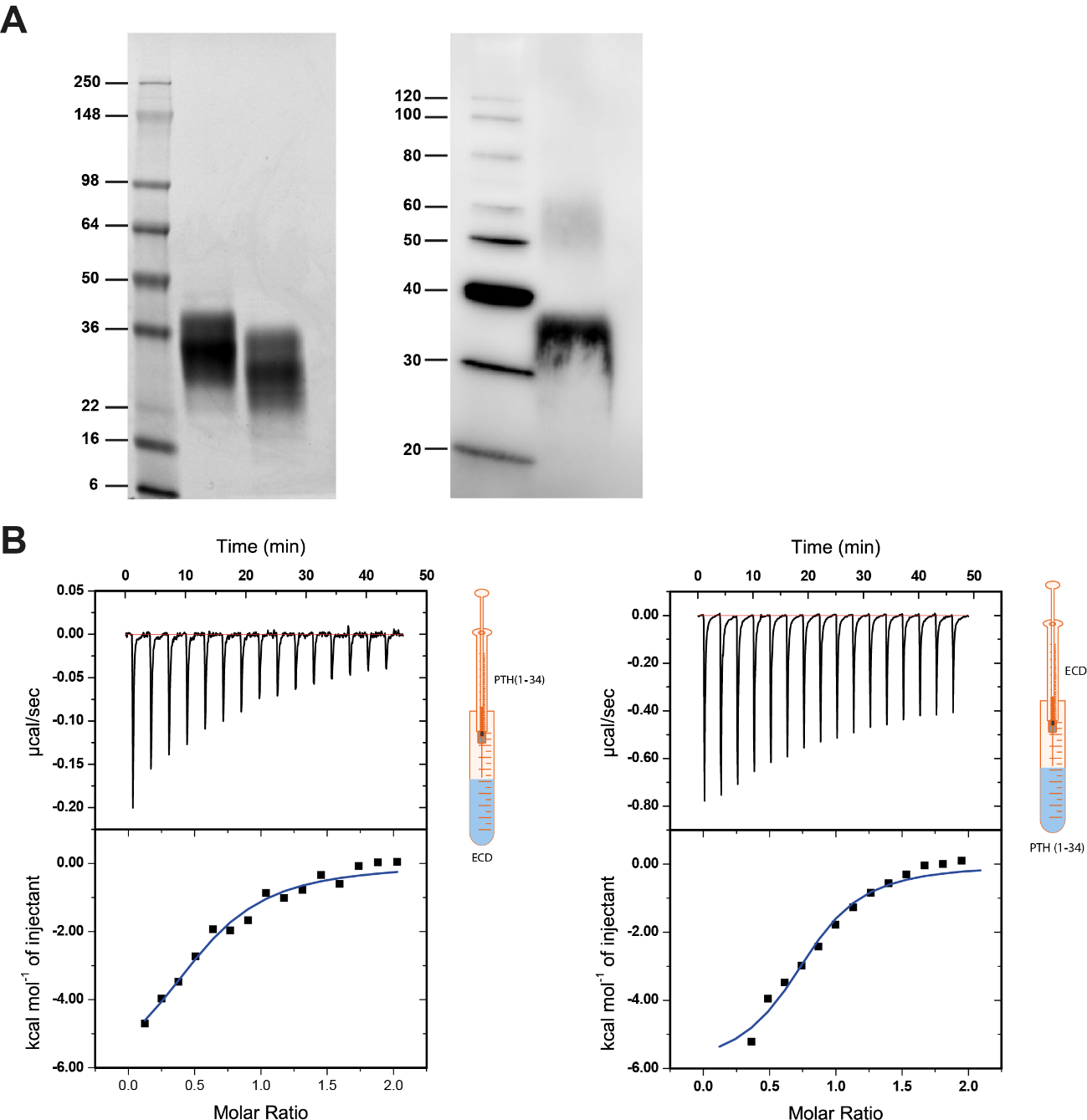
**

A) SDS-PAGE (L) and Western blotting (R) analysis of the pure PTH1R ECD. The protein was separated on a 4-20% Tris-glycine gel and the bands visualised using Coomassie Blue stain. The broad nature of the PTH1R ECD band is indicative of glycosylated protein. B) ITC analysis of the PTH1R ECD. In the left panel the ligand was titrated into a solution of the purified protein, while in the right panel the purified ECD was titrated into the ligand. The binding affinity of the ECD for PTH-(1-34) was approximated to 4 µM (titrating PTH (1-34) into PTH1R ECD) and 3.8 µM (titrating PTH1R ECD into PTH (1-34)).

## Supplementary Figure 2: Phage ELISA and cell surface binding of scFV-mFc.

**
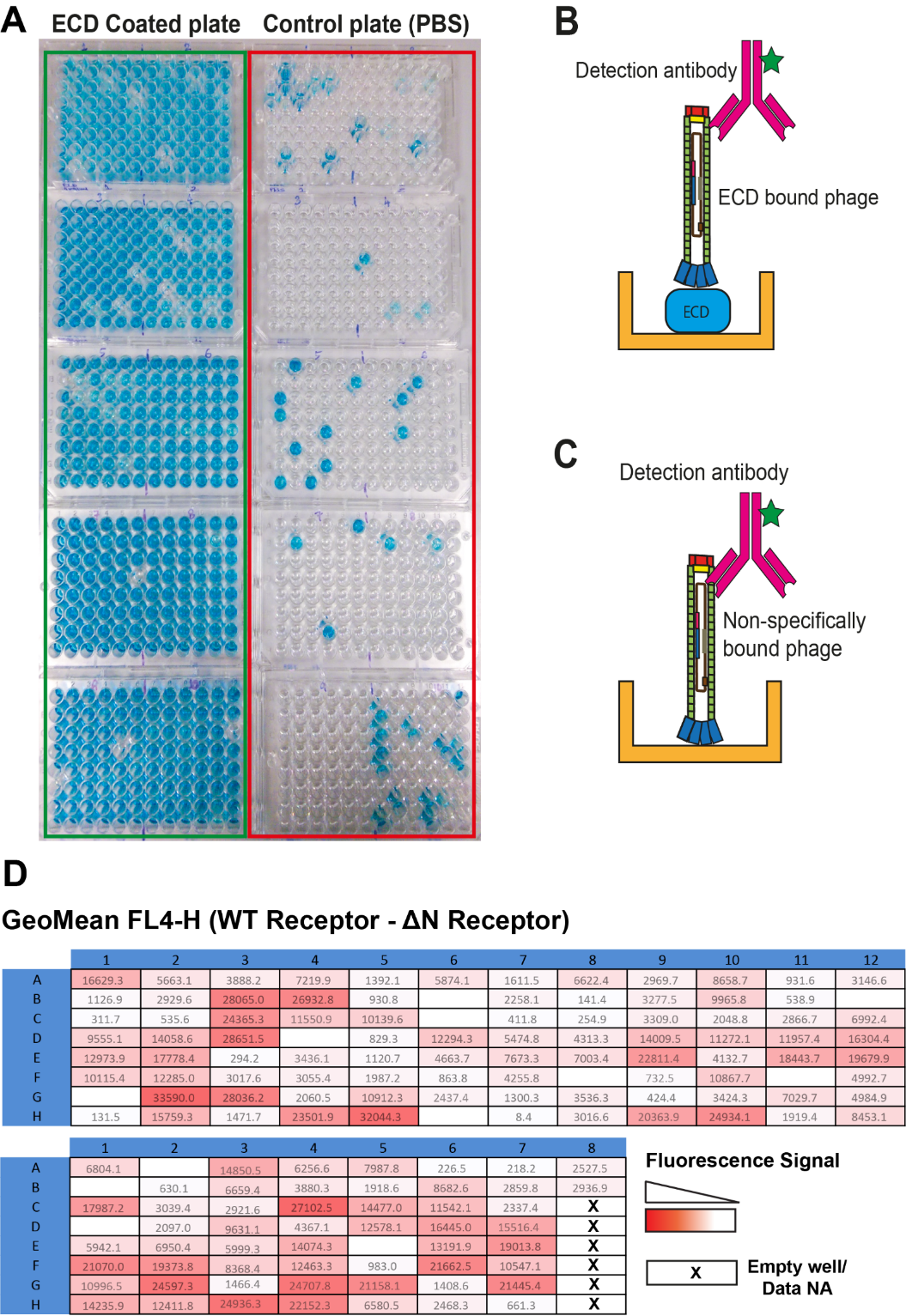
**

A) Phage ELISA plates with hits from three rounds of panning. B and C show schematic representations of specific and non-specific phage binding respectively. A total of 411 ECD specific clones were found after three rounds of panning against the purified ECD. D) ΔN-corrected mean fluorescence for scFvmFc TAP products binding to cells expressing WT receptor. The shades of red represent the intensity of the fluorescence signal from each well with darker shade representing higher fluorescence. Wells with no detectable fluorescence signal (white) suggest complete lack of binding to the full-length receptor. Cells with X represents empty wells.

## Supplementary Figure 3: SDS-PAGE analysis of selected scFvhFc


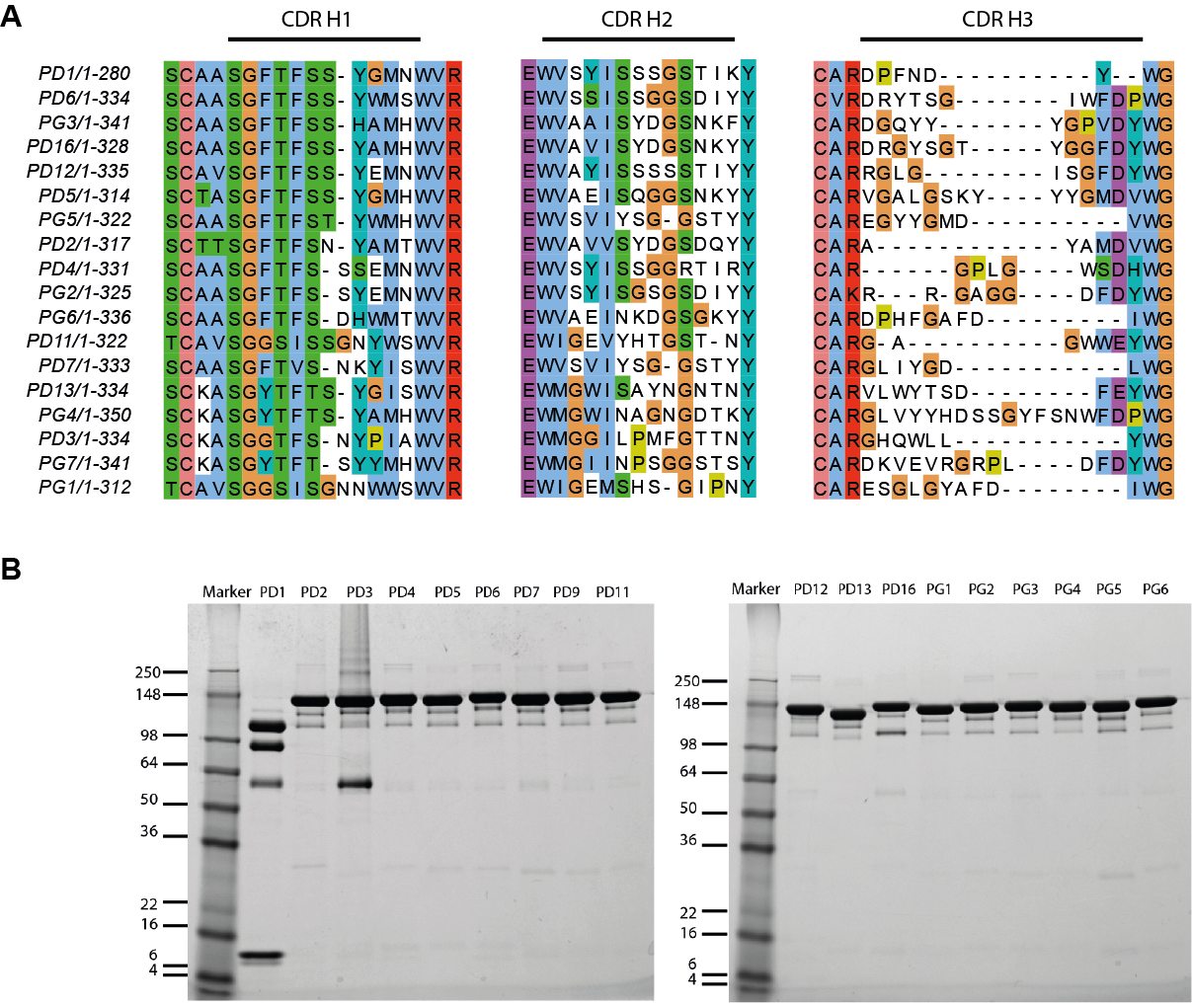


SDS-PAGE analysis was used to determine the purity of affinity (Protein A) purified scFvhFc samples.

## Supplementary Figure 4: Flow-cytometry analysis of the 18 scFvhFc clones


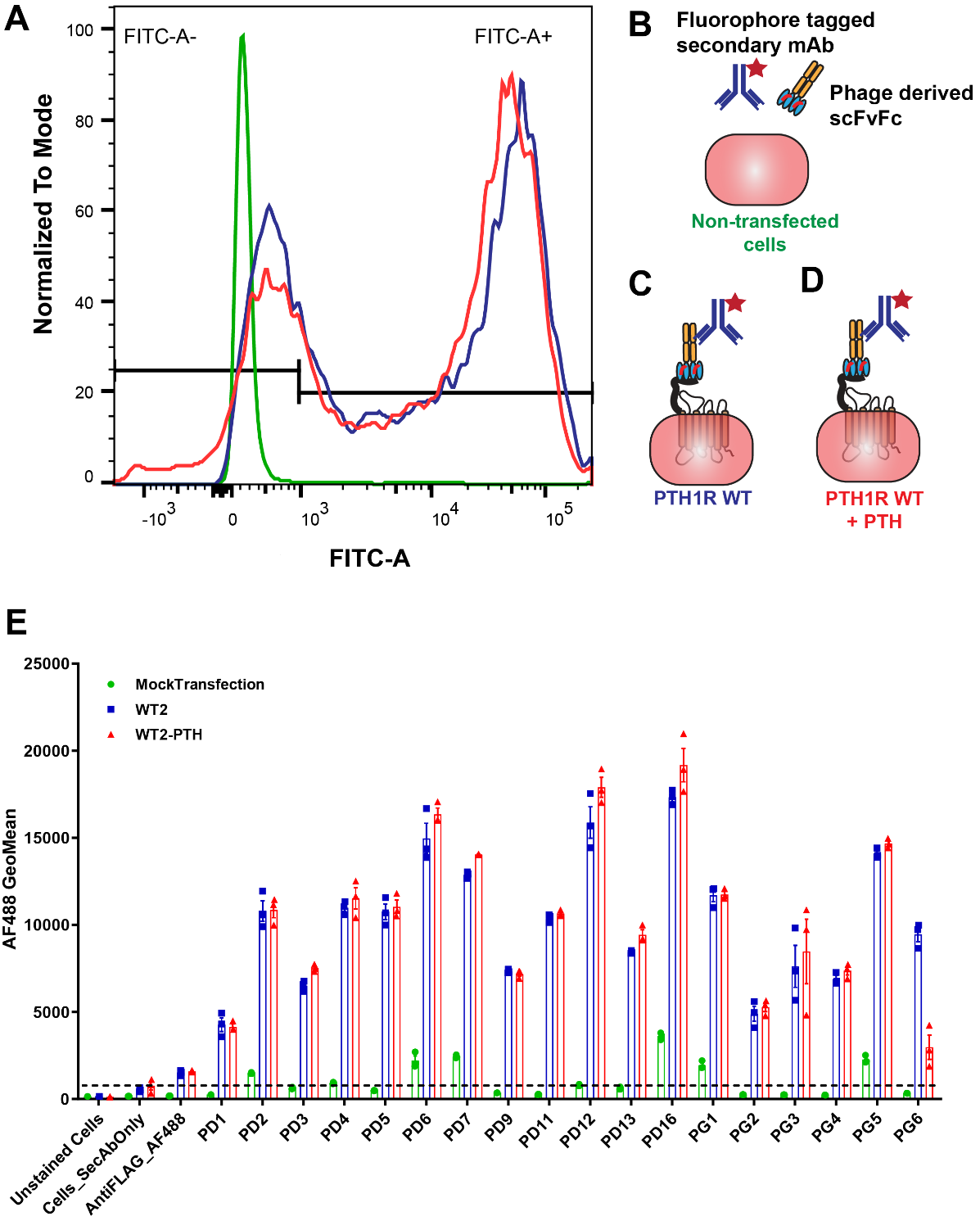


A) Overlay of representative flow cytometry data for PD9-scFvhFc binding to cells transfected with PTH1R WT2 receptor (cyan) (C) and non-transfected (green) cells (B). The overlay also shows the effect of PTH (1-34) addition to the WT2 expressing cells (red trace) (D). E) A plot summarising the geometric mean of fluorescence as shown by all selected ECD binding scFvhFc molecules. Each data point represents the geometric mean and standard deviation of a technical replicate. The black dashed line represents the fluorescence levels from cells stained with secondary antibody only.

## Supplementary Figure 5: PTH (1-34) concentration- response curves using PTH1R-expressing stable and parental CHOK-1 cells


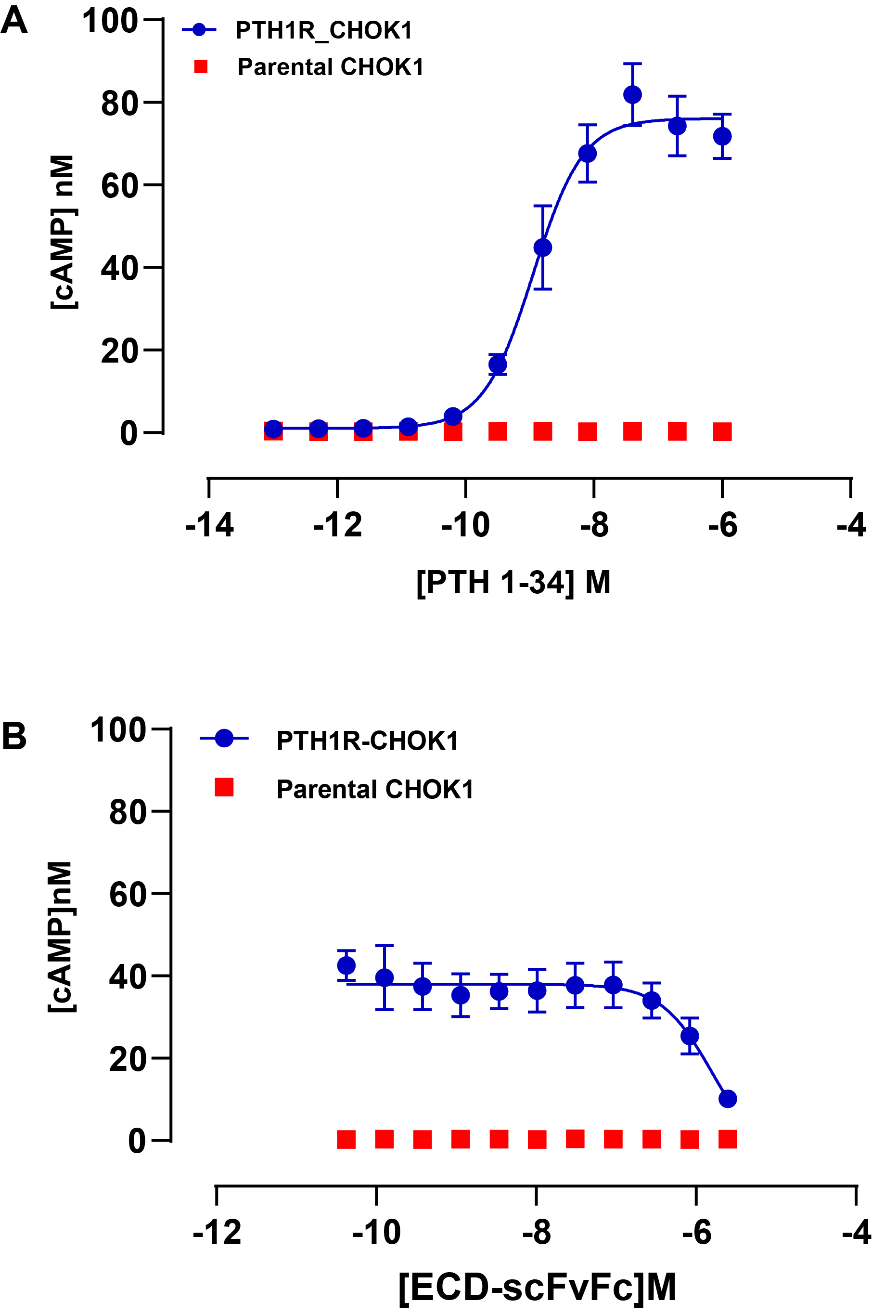


A) PTH (1-34) concentration response curve for CHOK-1 cells stably expressing PTH1R (blue) and parental CHOK-1 cell line (red). Stable cells show dose-dependent increase in cAMP production whereas no increase in cAMP was seen with parental cell-line. B) Effect of ECD-scFvhFc binding in PTH (1-34) mediated cAMP production was determined by incubating PTH1R-expressing stable cells (blue) and parental cells (red) with an ECD-scFvhFc concentration range and challenging cells with 0.4 nM of PTH (1-34). Only a slight decrease in cAMP production was detected at a very high ECD-scFvhFc concentration. Each data point represents means and SD of quadruplicate measurements.

## Supplementary Table 1

|  | n=1 | | n=2 | |  |  |
| --- | --- | --- | --- | --- | --- | --- |
|  | **K_D_ (nM)^b^** | **K_D_ (nM)^c^** | **K_D_ (nM)** | **K_D_ (nM)** | **Mean** | **Std.Dev** |
| Molecule^a^ | **Fc = 2-1** | **Fc = 3-4** | **Fc = 2-1** | **Fc = 3-4** |  |  |
| PD1 | 253.6 | 216.7 | 204.6 | 188.1 | 215.8 | 27.8 |
| PD2 | 1687 | 1488 | 1348 | 1307 | 1457.5 | 171.5 |
| PD3 | 1245 | 1003 | 1043 | 929.1 | 1055.0 | 135.2 |
| PD4 | >2000 | >2000 |  |  | ND^e^ | ND |
| PD5 | 1076 | 733.9 | 959 | 882.8 | 912.9 | 143.4 |
| PD6 | 1319 | 803.3 | 966.3 | 868.6 | 989.3 | 229.8 |
| PD7 | >2000 | >2000 |  |  | ND | ND |
| PD9 | 1230 | 926.5 | 1058 | 920.1 | 1033.7 | 145.5 |
| PD10 | 349.5 | 208.4 | 270.8 | 253.7 | 270.6 | 58.8 |
| PD12 (ECD-scFvhFc) | 4.551 | 3.784 |  |  | 4.2 | 0.5 |
| PD13 | 518.2 | 519.5 | 351.7 | 321.2 | 427.7 | 106.0 |
| PD16 | 746 | 608.2 | 395 | 356.7 | 526.5 | 183.5 |
| PG1 | 300.3 | 270.2 | 183.9 | 165.6 | 230.0 | 65.4 |
| PG2 | 48.46 | 44.95 | 41.79 | 39.03 | 43.6 | 4.1 |
| PG3 | 58.78 | 54.49 | 63.02 | 58.66 | 58.7 | 3.5 |
| PG4 | 381.2 | 329.3 | 310.2 | 281.1 | 325.5 | 42.1 |
| PG5 | 30.37 | 35.82 | 29.87 | 29.36 | 31.4 | 3.0 |
| PG6 | 1473 | 1423 | 902.7 | 825.6 | 1156.1 | 339.2 |

**^a^ Molecule ID;**

**^b^The measured affinity from flow cell 2-1 in nanomolar;**

**^c^The measured affinity from flow cell 3-4 in nanomolar;**

**^d^Model used to fit the kinetics data. In most cases 1:1 model gave accurate fitting.**

**^e^ ND represents not-determined**

## Supplementary Table 2

HDX data for ECD peptides (0.5 min HDX reaction). The p values depict the statistical significance of the % difference in deuterium uptake between the apo and ECD-scFvhFc condition.

| Colour Key: | ΔHDX/ Da | ΔHDX^b^/ % | | t-test | |  |  |  |
| --- | --- | --- | --- | --- | --- | --- | --- | --- |
|  | >1 | >5 | | p<0.001 | |  |  |  |
|  | 0.5-1 | 2-5 | | p<0.01 | |  |  |  |
| Sequence | | | average m/z (apo)^a^ | | ΔHDX^b^/ Da | | ΔHDX^c^/ % | T-Test |
| AYALVDADDV | | | 528.18 | | 0.17 | | 1.87 | 0.00415 |
| YALVDAD | | | 770.26 | | 0.30 | | 4.96 | 0.10902 |
| YALVDADD | | | 885.44 | | 0.28 | | 3.96 | 0.02557 |
| ALVDADD | | | 721.55 | | 0.23 | | 3.80 | 0.00784 |
| ALVDADDVM | | | 953.07 | | 0.59 | | 7.33 | 0.00107 |
| ALVDADDVMT | | | 1054.91 | | 1.04 | | 11.58 | 0.00068 |
| DVMTKEEQ | | | 492.18 | | 1.21 | | 17.27 | 0.00010 |
| DVMTKEEQIF | | | 622.55 | | 1.26 | | 14.02 | 0.00007 |
| VMTKEEQ | | | 434.48 | | 1.78 | | 29.61 | 0.00010 |
| VMTKEEQIF | | | 564.63 | | 1.19 | | 14.84 | 0.00005 |
| VMTKEEQIFL | | | 621.16 | | 1.26 | | 14.05 | 0.00007 |
| MTKEEQ | | | 767.78 | | 0.57 | | 11.31 | 0.38487 |
| TKEEQIF | | | 448.83 | | 0.93 | | 15.47 | 0.00023 |
| TKEEQIFL | | | 505.37 | | 0.91 | | 13.06 | 0.00015 |
| KEEQIFL | | | 908.15 | | 0.65 | | 10.76 | 0.00030 |
| EQIFLL | | | 763.41 | | 0.31 | | 6.22 | 0.00521 |
| IFLLHRAQAQC | | | 651.20 | | 1.11 | | 11.13 | 0.00020 |
| IFLLHRAQAQCEKRLKEVLQRPA | | | 689.25 | | 1.26 | | 6.01 | 0.00138 |
| LLHRAQAQCEKRLKEVLQRPA | | | 499.60 | | 0.66 | | 3.50 | 0.01321 |
| LHRAQAQCEKRLKEVLQRPA | | | 477.00 | | 0.66 | | 3.68 | 0.00513 |
| EKRLKEVLQRPA | | | 491.22 | | 0.42 | | 4.20 | 0.00482 |
| KRLKEVLQRPA | | | 671.78 | | 0.43 | | 4.80 | 0.00989 |
| VLQRPA | | | 685.49 | | 0.01 | | 0.32 | 0.26209 |
| VLQRPAS | | | 773.45 | | 0.04 | | 0.80 | 0.21780 |
| VLQRPASIM | | | 510.57 | | 0.54 | | 7.68 | 0.00478 |
| RPASIMESDKGW | | | 460.34 | | -0.08 | | -0.75 | 0.15878 |
| SIMESDKGWTSA | | | 659.84 | | 0.37 | | 3.35 | 0.00497 |
| SIMESDKGWTSAS | | | 703.81 | | 0.29 | | 2.41 | 0.00035 |
| SIMESDKGWTSASTSGKPRKDKA | | | 827.36 | | 0.60 | | 2.85 | 0.00786 |
| IMESDKGWTSASTS | | | 754.69 | | 0.27 | | 2.04 | 0.00492 |
| MESDKGWTSASTSGKPRKDKA | | | 570.36 | | 0.54 | | 2.85 | 0.03243 |
| ESDKGWTSA | | | 985.20 | | 0.17 | | 2.09 | 0.11911 |
| ESDKGWTSAS | | | 536.96 | | 0.13 | | 1.49 | 0.08893 |
| ESDKGWTSASTS | | | 631.37 | | 0.25 | | 2.31 | 0.03840 |
| SGKLYPESE | | | 507.39 | | 0.14 | | 1.93 | 0.15955 |
| SGKLYPESEE | | | 572.22 | | 0.20 | | 2.46 | 0.00243 |
| SGKLYPESEED | | | 629.60 | | 0.16 | | 1.80 | 0.02513 |
| SGKLYPESEEDKEAPTGSRY | | | 752.16 | | 0.65 | | 3.82 | 0.00719 |
| GKLYPESE | | | 463.31 | | 0.05 | | 0.79 | 0.01187 |
| GKLYPESE | | | 926.05 | | 0.00 | | 0.06 | 0.98333 |
| DKEAPTGSRYRGRPCL | | | 453.59 | | 0.01 | | 0.09 | 0.90453 |
| PEWDHILCWPLGAPGEV | | | 960.71 | | 0.07 | | 0.54 | 0.63870 |
| EVVAVPCPD | | | 466.00 | | 0.53 | | 8.79 | 0.00175 |
| VVAVPCPD | | | 401.60 | | 0.17 | | 3.35 | 0.28388 |
| VVAVPCPDY | | | 963.57 | | 0.33 | | 5.42 | 0.05316 |
| VAVPCPDY | | | 864.62 | | 0.27 | | 5.30 | 0.00381 |
| VPCPDY | | | 694.09 | | 0.11 | | 3.57 | 0.00043 |
| IYDFNHKGHA | | | 601.81 | | -0.07 | | -0.76 | 0.02714 |
| FNHKGHA | | | 811.00 | | -0.03 | | -0.55 | 0.37157 |
| YRRCDRNGSW | | | 657.78 | | 0.00 | | -0.03 | 0.93856 |
| CDRNGSWELVPGHNRTWAN | | | 1267.49 | | 0.92 | | 5.44 | 0.02679 |
| ELVPGHD | | | 767.36 | | -0.12 | | -2.40 | 0.01419 |
| ELVPGHDRTWAD | | | 699.39 | | -0.07 | | -0.65 | 0.03113 |
| ELVPGHDRTWADY | | | 521.20 | | 0.22 | | 2.03 | 0.23095 |
| PGHDRTWAD | | | 528.61 | | -0.05 | | -0.70 | 0.13731 |
| PGHDRTWADYSE | | | 718.47 | | -0.14 | | -1.37 | 0.17571 |
| VKFLTDETRE | | | 621.71 | | -0.21 | | -2.30 | 0.00301 |
| FLTNETREREVF | | | 1222.00 | | 0.42 | | 3.79 | 0.08451 |
| LTDETRE | | | 433.67 | | -0.15 | | -2.42 | 0.04911 |
| LTDETREREVF | | | 699.04 | | -0.17 | | -1.68 | 0.02252 |
| TDETRE | | | 752.89 | | -0.27 | | -5.49 | 0.01438 |
| ETREREVF | | | 534.70 | | -0.54 | | -7.78 | 0.00368 |

^a^Most intense charge state used as representative per peptide.

^b^ Difference in HDX (ΔHDX)= [average m/z (apo) – average m/z (bound)] x peptide charge state

^c^%ΔHDX = difference in Da / number of exchangeable backbone amide protons in peptide (excluding N-terminal residue)

## Supplementary Methods

### Phage rescue and helper phage infection

Bacterial colonies were harvested by scraping into 10 ml 2TY broth and the suspension centrifuged at 3000 x g for 10 minutes. A new 10 ml culture of 2YT broth supplemented with 1% glucose and 100 µg/ml Carbenicillin (2YTAG) was started using inoculation from these cells (starting OD_600_ = 0.1) and grown with shaking at 37°C to OD_600_ = 0.5-0.8, when helper phage was added at a multiplicity of infection (MOI) of 20. The tubes were incubated at 37°C for 1 h without agitation and the cells collected by centrifugation at 3500 x g for 10 min. To facilitate the release of phage particles, the bacterial cell pellet was re-suspended in 10 ml 2TY broth (supplemented with 100 µg/ml carbenicillin and 50 µg/ml kanamycin) and incubated overnight at 30°C with shaking at 300 rpm. The culture was centrifuged at 3000 x g for 10 minutes to pellet the cells. The supernatant, containing the progeny phage particles, was transferred to a separate tube and centrifuged at 12,000 x g for 20 min and the resulting supernatant transferred to a fresh 50 ml polypropylene tube. The phage particles were precipitated by adding 2 ml phage precipitation solution (20% w/v Polyethylene Glycol 8000, 2.5 M NaCl) to the supernatant and incubating the solution for 1 h on ice. The precipitated phage particles were collected by further centrifuging the tubes at 3500 x g for 10 min and the resulting pellet re-suspended in 1 ml of PBS. After the third round of panning, single isolated bacterial colonies were consolidated into 48-well culture blocks, referred to as master plates for monoclonal antibody screening.

### Phage ELISA to determine enrichment.

The phage particles from overnight monoclonal helper phage rescues were blocked using phage blocking solution and added to plates coated with either the ECD or PBS and incubated at room temperature for 1 h. The plates were washed three times using PBST and the Anti-M13 Phage-HRP conjugated antibody (GE Healthcare) at a final dilution of 1: 5000 (in PBS + 3% BSA) added to each well prior to a further incubation at room temperature for 1 h. Following a final wash, 50 µl of 3, 3′, 5, 5′-Tetramethylbenzidine (TMB) reagent was added and the absorbance at 630 nm measured using a plate reader. DNA was prepared from colonies with detectable ECD binding and sequenced. The sequence diversity of the heavy chain variable domain’s complementarity determining region 3 (V_H_ CDR3) was initially used to screen for unique binders.

### Analysis of the HDX data

Default PLGS processing parameters included: Low Energy Threshold: 100 counts; Elevated Energy Threshold: 50 counts; Elution Start Time: 1 minute; Elution End Time: 9 minutes; Intensity Threshold: 500 counts. PLGS Workflow search parameters included: Minimum fragment ions matched: 3; Primary digest reagent: non-specific; Variable glycosylation modification: N-linked; False discovery rate: 4. Data were exported into DynamX and peptide assignments filtered further as follows: Minimum intensity: 1000; Maximum sequence length: 25; Minimum products per amino acid: 0.2; Maximum MH+ error: 10 ppm. After processing, all assignments were interrogated manually, and only peptides with a suitable signal to noise ratio and unambiguous peak-picking were taken forward.
